# Appropriate Reference Parameters for the Evaluation of Elemental Analysis Data from Biomedical Specimens

**DOI:** 10.6028/jres.093.063

**Published:** 1988-06-01

**Authors:** Venkatesh Iyengar, Dietrich Behne

**Affiliations:** NBS/USDA, Gaithersburg, MD; HMI, Berlin, F.R. Germany

Studies on the elemental composition of biological systems can be divided into four stages: experimental design, collection of valid samples, chemical analysis and data evaluation (and interpretation). Each of these steps is important for the overall success of an investigation. In our opinion, one aspect of data evaluation and interpretation of trace element studies, namely relating the elemental analysis data to a meaningful base, has not received adequate attention. In many cases this has resulted in wrong conclusions being reached, even if the elemental analysis has been carried out correctly. This frequently happens when the biological material consists of several components with different elemental content, and the ratio of these components differs from sample to sample [1].

Elemental analysis in bone samples is an example of this phenomenon, as several elements are unevenly distributed between the different bone compartments. The fluorine content of the trabecular bone, for instance, is greater than that in the compact substance by a factor of 3 [1]. As the ratio of spongy to compact substance varies along the bone, different fluorine contents are estimated for the whole sample, according to where the sampling has been carried out, although no changes in the fluorine levels in the different bone tissues have occurred. Forbes et al. [2] have reported a similar phenomenon for lead within a rib; expressed on the basis of ash, Pb content decreases as the distance from the costochondral junction increases. This has been attributed to the increased ash content of the samples as the fraction of marrow changes. Therefore, it is necessary to specify the location of the bone section analyzed and its marrow content.

Similarly, the results of elemental analysis of whole blood may depend on the percentage of red blood cells in the sample [1]. This should always be considered when the elemental content is much higher in erythrocytes than in the blood plasma, as for instance, in the case of iron and lead. If not, changes in the hematocrit, which can occur as a function of several factors (such as pregnancy, age or disease) may lead to false conclusions being drawn about the quantities of these elements in the body.

Other examples of this source of error are the changes in the age distribution of erythrocytes after blood loss [3], which results in a higher zinc content of the red blood cells, changes in the water content of serum samples according to the body position during sampling (recumbent or upright) [4], or changes in the amount of fat or residual blood in a tissue, all of which can lead to alterations in the content of several elements.

Errors can also occur by redistribution of elements or water in certain organs due to the interruption of metabolic processes. For instance, significant changes in the weight of the whole organ can occur under post-mortem conditions [5]. This can lead to great differences in elemental concentrations, depending on whether they are related to the wet or the dry weight of the material. In such cases, it is necessary to know the total weight of the organ, as well as its water content, to be able to account for changes in elemental concentrations. It is obvious that meaningful data interpretation is not possible without this knowledge.

Many of the errors alluded to above can be avoided if one does not express the analytical value only in the usual way of amount per volume or weight of the sample, but also relates the data to other parameters such as the number of certain cells or the content of a protein. This enables changes in the tissue composition to be taken into account. For instance, in whole blood analysis errors due to changes in the hematocrit can be excluded, if the amount of hemoglobin, which reflects the number of red blood cells, is used as a base for expressing elemental concentrations.

Some examples of relevant parameters needed to evaluate elemental analysis data are shown in table 1. In table 2 additional features are listed, which should also be checked when elemental data are measured in biological materials.

The authors recognize that it is difficult to recommend a most suitable base, since this generally depends on the type of study and on the problems to be investigated. However, one can improve the present situation by paying adequate attention to documentation of supplemental information on relevant parameters and by including them in scientific publications. This would facilitate a meaningful intercomparison of results from different investigations.

In conclusion, it is important to present results from biological trace element investigations in an unambiguous way. A multidisciplinary approach is essential throughout the whole investigation. Only then will it be possible to eliminate the influence of presampling factors [6], to obtain biologically and analytically valid specimens for analysis [7,8] and to choose the most appropriate parameters for data evaluation and interpretation.

## Figures and Tables

**Table 1:** Parameters for evaluation of elemental analysis data in biological specimens^a^

Specimens	Parameters
Body fluids	
Serum, plasma	Protein, water
Whole blood	Hematocrit
Urine	24 h volume, creatinine
Sweat	Volume
Amniotic fluid	Meconium, cells
Milk	Average daily output, fat content
Semen	Sperm count
Bile	Volume
Cells	
RBC, WBC, Platelets, Spermatozoa	Cell number and Haemoglobin (for RBC)
Soft tissues	
Organ samples	Whole weight of the organ
Placenta	Age of the placenta, total weight.
Hard tissues	
Bone	Percentage marrow, ratio spongy/compact, Ca/P ratio.
Teeth	Ratio pulp/enamel
Hair	Distance from surface
Diets^b^	24 h collection weights, average body weights of subjects
Feces^c^	24 h collection weights, average body weights of subjects

a Specific Gravity, dry/wet weight ratios, common for all fluids, moisture content common for both hard and soft tissues, diets and feces.

b Expressed as intake/day.

c Expressed as excretions/day.

**Table 2:** Checklist of essential features related to elemental analysis of biological systems

Specimen	Required information
Plasma	Amount and type of anticoagulant and its chemical purity
Serum	Body position during sampling, hemolysis status
Milk	Days past partum, specific fraction (hindmilk, foremilk) if entire volume not collected
Cells	Viability, (cell age), amount. Type and purity of stabilizer used, trapped plasma
Soft tissues	Residual blood, decidual tissue while handling placenta, biopsy or autopsy
Hard tissues	
bone	Biopsy or autopsy, renal function status sampling location
hair and nail	Origin and washing procedures for hair and nail
Diets	Proximate composition, caloric energy
Feces	Occult blood
